# A Supramolecular Photosensitizer System Based on Nano-Cu/ZIF-8 Capped with Water-Soluble Pillar[6]arene and Methylene Blue Host–Guest Complexations

**DOI:** 10.3390/molecules26133878

**Published:** 2021-06-25

**Authors:** Chenhao Hu, Yueyuan Yu, Shuang Chao, Huidan Zhu, Yuxin Pei, Lan Chen, Zhichao Pei

**Affiliations:** 1 Key Laboratory of Natural Products & Chemical Biology, College of Chemistry and Pharmacy, Northwest A&F University, Yangling 712100, China; huchenhao0620@163.com (C.H.); gongt118410@163.com (Y.Y.); chaoshuang@nwafu.edu.cn (S.C.); peiyx@nwafu.edu.cn (Y.P.); 2 Center of College of Science & Technology, Hebei Agricultural University, Huanghua 061100, China; kyrazj1023@163.com

**Keywords:** PDT, CDT, host–guest complexation, pillar[6]arene, combination therapy

## Abstract

Photodynamic therapy (PDT) as a safe, non-invasive modality for cancer therapy, in which the low oxygen and high glutathione in the tumor microenvironment reduces therapeutic efficiency. In order to overcome these problems, we prepared a supramolecular photosensitive system of O_2_-Cu/ZIF-8@ZIF-8@WP6–MB (OCZWM), which was loaded with oxygen to increase the oxygen concentration in the tumor microenvironment, and the Cu^2+^ in the system reacted with glutathione (GSH) to reduce the GSH concentration to generate Cu^+^. It is worth noting that the generated Cu^+^ can produce the Fenton reaction, thus realizing the combination therapy of PDT and chemodynamic therapy (CDT) to achieve the purpose of significantly improving the anti-cancer efficiency.

## 1. Introduction

As a safe and non-invasive method, PDT has been used in the treatment of numerous diseases. Especially in cancer treatment, PDT can not only reduce the recovery time of delicate surgical treatment, but also effectively kill drug- and radiation-resistant tumor cells, making it a new approach to replace traditional therapy [1,2]. As the core of PDT, photosensitizers (PSs) interact with oxygen molecules in tumor tissues under the stimulation of light to release reactive oxygen species (ROS), which can oxidize cell components and lead to cell necrosis and/or apoptosis [3,4,5]. However, low oxygen and high glutathione in the tumor microenvironment influences PSs to produce ROS, which leads to a reduction in the therapeutic efficiency [6,7,8]. In order to improve the problems encountered by PSS in PDT application, chemodynamic therapy (CDT), as a recent and emerging treatment method, produces hydroxyl radicals through the Fenton-like or Fenton reaction with hydrogen peroxide in cancer cells, and then triggers the apoptosis of cancer cells, which is often combined with PDT to improve therapeutic effect [9,10]. As a kind of synthetic hybrid materials, MOFs have shown great application potential in gas storage, chemical separation, catalysis, sensing, and drug delivery due to their high porosity [11,12]. In particular, supramolecular hybrid MOFs can not only effectively load PSs (to avoid dark toxicity, hydrophobicity, and photobleaching of the PSs), but also use metal ions in their structure to Fenton react with hydrogen peroxide in tumor cells [13,14].

**Scheme 1:**
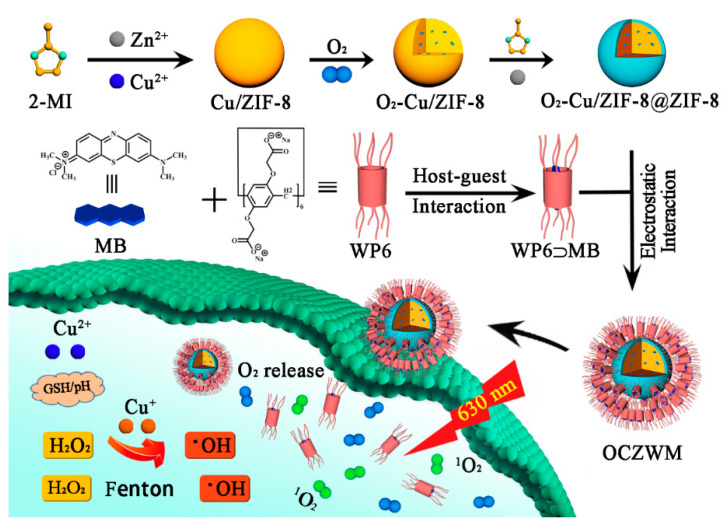
Schematic of the construction of supramolecular photosensitizer system based on nano-Cu/ZIF-8@ZIF-8 capped with the host–guest complexation between WP6–MB.

Consequently, the development of a new supramolecular PSs-MOFs carriers to maintain or even improve the efficiency of PDT can not only overcome the photobleaching and dark toxic effects of PSs, but also the combination therapy of PDT and CDT can be achieved. Zeolitic imidazolate frameworks-8 (ZIF-8) is an important class of MOFs, which has great potential in the construction of supramolecular drug delivery systems due to its stability in neutral, alkaline aqueous solutions and rapid decomposition in acidic solutions [15,16]. In addition, pillar[n]arenes, as a novel macrocyclic host molecule, can be used to construct nano-drug carriers through host-guest interaction [17,18], thus improving the solubility and stability of drugs [19,20,21]. Recently, based on the coordination between water-soluble carboxylated pillar[6]arene (WP6) and ZIF-8@DOX, we have developed a supramolecular targeted drug hybrid material with good dispersion efficiency, and host–guest complexation of WP6 and G as necessary modifiers to improve it0s water dispersion and give it target properties [22,23]. Herein, we design and synthesize Cu/ZIF-8@ZIF-8 nano carrier to adsorb O_2_ and participate in a Fenton reaction with Cu^2+^ achieving CDT therapy. Thereafter, a supramolecular photosensitive system was prepared based on Cu/ZIF-8@ZIF-8 nano carriers capped with the host–guest complexation WP6-methylene blue (WP6-MB), while the WP6-MB complexations can prolong the ROS production time of MB under light irradiation, overcome photobleaching, and can also realize CDT/PDT combination therapy (see Scheme 1).

## 2. Results and Discussion

**Figure 1:**
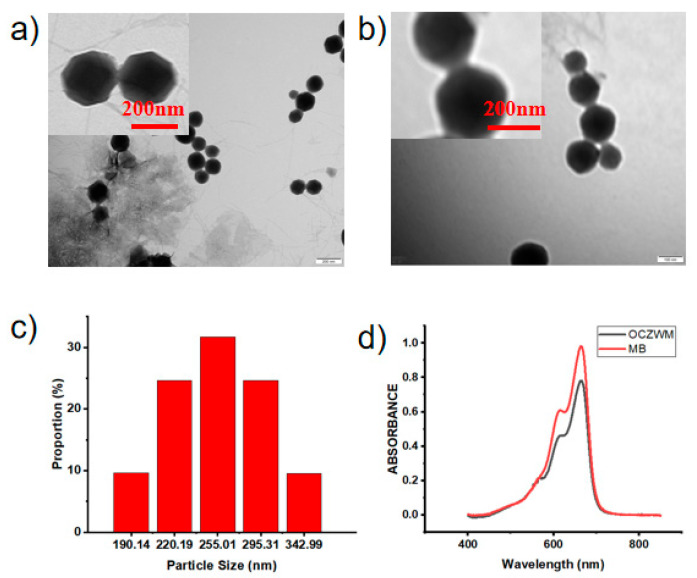
TEM images of (**a**) Cu/ZIF-8@ZIF-8 and (**b**) Cu/ZIF-8@ZIF-8@WP6–MB. The scale bar is 200 nm. Inset: partial enlarged images. The scale bar is 200 nm. (**c**) DLS data of Cu/ZIF-8@ZIF-8@WP6–MB. (**d**) UV-Vis spectra of Cu/ZIF-8@ZIF-8@WP6–MB and MB aqueous solution at room temperature.

WP6–MB host–guest complexation was synthesized according to the published procedure [22,24,25], which was fully characterized by ^1^H NMR (Figures S1 and S2 in the Supporting Information). Cu/ZIF-8@ZIF-8 was first synthesized according to the published procedure [23,26]. The scanning electron microscopy (SEM) and transmission electron microscopy (TEM) showed that Cu/ZIF-8@ZIF-8 had typical hexagon morphology, with a diameter of approximately 190–290 nm (Figure 1a,b and Figure S4). In addition, TEM images showed that the obtaining Cu/ZIF-8@ZIF-8@WP6–MB had an obvious fuzzy edge compared with that of the obtaining Cu/ZIF-8@ZIF-8, which can be ascribed to the assembly of WP6–MB (Figure 1c). In order to further illustrate the homogeneity of ZIF materials in this design, we carried out an X-ray diffraction (Figure S4). The Cu/ZIF-8@ZIF-8@WP6–MB and MB UV-Vis spectra also demonstrated the WP6–MB capped Cu/ZIF-8@ZIF-8 through assembly (Figure 1d). In addition, the zeta potentials of OCZWM and ZIF were measured to prove the complexation of ZIF with WP6–MB (Figure S5). As the molar ratio of WP6 and MB is 1:1, and the mass ratio of WP6–MB and ZIF is 1:1, after calculation, it can be concluded that the loading rate of MB is 8.20% wt.

**Figure 2:**
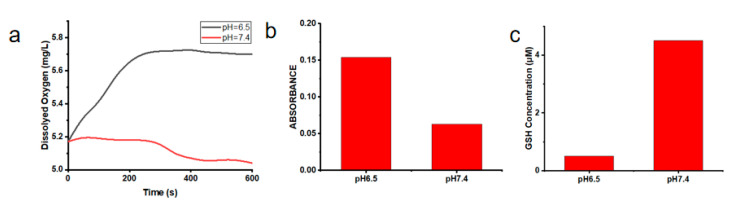
(**a**) the O_2_ content under the solution of OCZWM in pH = 6.5 and pH = 7.4. (**b**) The amount of ROS produced by OCZWM in pH = 6.5 and pH = 7.4. (**c**) The GSH content under the solution of OCZWM in pH = 6.5 and pH = 7.4.

To further demonstrate the loaded O_2_ ability of Cu/ZIF-8@ZIF-8@WP6–MB, the O_2_ content in the solution of OCZWM was measured under pH = 6.5 and pH = 7.4. As shown in Figure 2a, under acidic conditions, O_2_ content in the OCZWM solution increased with time. However, under neutral conditions, there was no significant change in the O_2_ content of the solution, indicating that the OCZWM had good O_2_ loading and pH responsiveness. It is worth noting that the desorption properties of ZIF-8 in acidic environment led to the collapse of the system and the decomposition of oxygen, which is independent of the supramolecular structure. Then, we used an ROS probe to investigate the ROS content produced by the solution of OCZWM at 630 nm light irradiation under pH = 6.5 and pH = 7.4 (Figure 2b). The results show that the amount of ROS produced by OCZWM under neutral conditions is significantly lower than that under acidic conditions, which also verifies that the nanoparticles have good pH responsiveness. It also indicates that the O_2_ released by nanoparticles in acidic environment can improve the production ROS by MB and the photosensitive efficiency. In addition, we utilized a GSH kit to detect the consumption of GSH by Cu^2+^ under pH = 6.5, pH = 7.4 conditions (Figure 2c). Obviously, due to the release of Cu^2+^ from the decomposition of nanoparticles in acidic solution, Cu^2+^ and GSH are reduced to form Cu^+^, resulting in a significant decrease in the concentration of GSH (see Figure 2c). It is worth noting that Cu^+^ is more prone to the Fenton reaction than traditional Fe^2+^ under the acidic conditions, and its ability to produce ·OH is stronger, which proves OCZWM to have a good CDT capacity.

**Figure 3:**
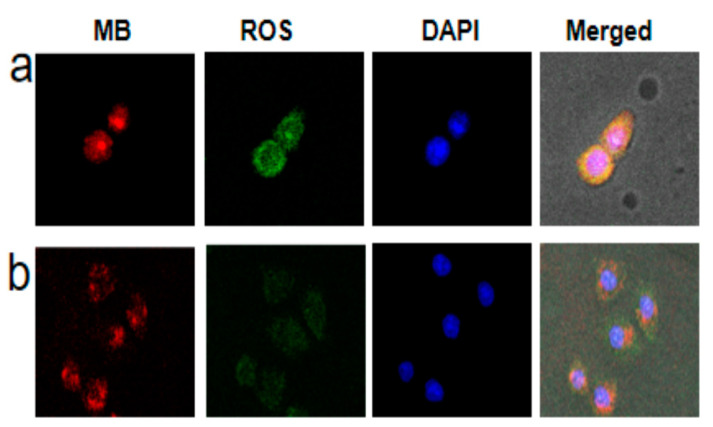
CLSM of HepG2 cells incubated with OCZWM (**a**) and Cu/ZIF-8@ZIF-8@WP6–MB (**b**). The scale bar is 50 µm. (**b**) the GSH content under the OCZWM, Cu/ZIF-8@ZIF-8@WP6–MB, MB, control.

Next, we investigated the feasibility of OCZWM as a PDT/CDT nanocarrier in cells. Firstly, taking the HepG2 cells as models, the cell membrane permeability and cell internalization of OCZWM were detected by confocal laser scanning microscopy (CLSM) (Figure 3a). The results showed that significant red fluorescence appeared in the cytoplasm after 4 h. Thereafter, we incubated 10 μm OCZWM with HepG2 cells for 4 h, and compared with or without light irradiation for 40 min, using DCFH-DA as the probe molecule to determine the production of ROS in the cells. As shown in Figure 3b, it can be clearly observed that after light irradiation, green fluorescence of DCFH-DA can be produced, proving that OCZWM can be used in living cells.

**Figure 4:**
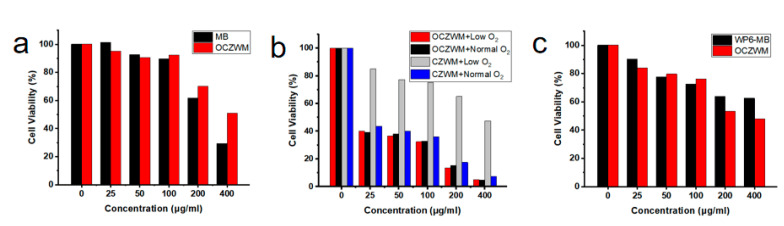
Relative cell viability of (**a**) HL7702 cells and (**b**) HepG2 cells after treatment with OCZWM, Cu/ZIF-8@ZIF-8@WP6–MB at different concentrations. (**c**) Relative cell viability of HepG2 cells after treatment with OCZWM, WP6–MB at different concentrations.

To gain further insight, the cell viability of OCZWM was carried out through MTT assay using HL7702 cells. As shown in Figure 4a, without light irradiation, OCZWM showed reduced dark toxicity compared to free MB with HL7702 cells. To provide further evidence of the anticancer attributes, the cell viability of OCZWM was carried out through MTT assay using HepG2 cells. Cu/ZIF-8@ZIF-8@WP6–MB were used as control groups. As shown in Figure 4b, under the condition of normal O_2_, the inhibition rates of OCZWM and Cu/ZIF-8@ZIF-8@WP6–MB on HepG2 cells were, in practical terms, the same. However, under the low O_2_ condition, the inhibitory rate of OCZWM on HepG2 cells did not significantly change, while the inhibitory rate of Cu/ZIF-8@ZIF-8@WP6–MB on HepG2 cells significantly decreased. This indicates that the OCZWM is released O_2_ in the tumor cells, improving the photodynamic therapy effect of MB. Moreover, it was found that, without light irradiation, the inhibition rates of OCZWM were higher than that of WP6–MB due to the CDT (Figure 4c).

## 3. Materials and Methods

All reagents were purchased from commercial suppliers and used without further purification unless specified. Water used in this work was triple distilled. NMR spectra were recorded on a Avance neo 400 MHz Spectrometer, with working frequencies of 400 MHz for ^1^H. Absorption spectra were collected by using a Shimadzu 1750 UV-visible spectrometer (Kyoto, Japan). Dynamic Light Scattering (DLS) data were obtained by Nano-2s ZEN3600. The confocal laser microscope (CLSM) data were acquired using a CLSM (Andor REVOLUTION WD). The power of light is 25 mW/cm^2^ at 630 nm. TEM images were obtained from FEI TECNAI G2 SPIRIT BIO. SEM images were obtained from Nano SEM-450. Flow cytometry data were obtained from BD FACSAria™ III.

### 3.1. General Procedure for Cell Culture

HL7702 and HepG2 cells were cultured at 37 °C and 5% CO_2_ in Dulbecco’s modified Eagle’s medium (DMEM, containing 10% fetal bovine serum (FBS) and 1% penicillin/streptomycin). The cells were fused with trypsin (0.5% *w*/*v* PBS) and separated. Cells were re-suspended in DMEM containing 10%FBS at a concentration of 1 × 10^4^ cells/mL.

### 3.2. Synthesis and Characterization of the Compounds WP6

WP6 was synthesized according to the literatures (S1–S3) and the ^1^H NMR spectrum (see Figure S1). ^1^H NMR (400 MHz, D_2_O) δ 6.61 (s, 12H), 4.05 (s, 24H), and 3.84 (s, 12H).

### 3.3. General Procedure for OCZWM

Cu/ZIF-8 was synthesized according to the literatures [22]. The DLS and SEM of Cu/ZIF-8 were shown in Figures S3 and S4. The Cu/ZIF-8 prepared above was vacuum-dried to remove the methanol in the pores, and then was dispersed in methanol again for later use after being pumped with O_2_ for 3 days. Then, ZIF-8 shells were uniformly grown around the Cu/ZIF-8 and the product (OCZ) was collected by centrifugation. Finally, WP6 assembled host–guest with MB in water (molar ratio 1:1) and the WP6–MB combined with OCZWM by coordination (mass ratio 1:1). The final products O_2_-Cu/ZIF8@ZIF-8@WP6–MB (OCZWM) were obtained by centrifugation.

## 4. Conclusions

In conclusion, we successfully developed a novel supramolecular photosensitizer system based on nano-Cu/ZIF-8 capped with the water-soluble pillar[6]arene and methylene blue host–guest complexations. The resulting OCZWM possessed excellent O_2_ load capacity and pH-sensitive release property. Flow cytometry and CLSM studies showed that OCZWM could be taken up by HepG2 cells and release MB efficiently. As a result, cell cytotoxicity measurements demonstrated that OCZWM in low O_2_ exhibited good toxicity for hepatoma cancer cells. Furthermore, the in vitro evaluation of CDT tests towards HepG2 cells have shown that OCZWM can consume GSH and significantly improve the efficiency for PDT compared with that of WP6–MB. Therefore, this work provides a good example of rational design of supramolecular photosensitizer system, which opens an efficient pathway for PDT/CDT combination therapy.

## Data Availability

The data presented in this study are available on request from the corresponding author.

## Supplementary Materials

The following are available online. Figure S1: ^1^H NMR spectrum (400 MHz, D_2_O) of WP6., Figure S2: ^1^H NMR spectra (400 MHz, D_2_O, 298 K): (a) WP6 (10.00 mM), (b) WP6:MB = 1:1; (c) MB (10.00 mM)., Figure S3: DLS data of Cu/ZIF-8., Figure S4: PXRD pattems of different materials, Figure S5: Zata potential studies of OCZWM, Figure S6: SEM images of Cu/ZIF-8 (a), Cu/ZIF-8@ZIF-8. The scale bar is 2 μm.

## Sample Availability

Samples of the compounds, carboxylated pillar[6]arene and the nanoparticles are available from the authors.
