# Supplementary material for: A Supramolecular Photosensitizer System Based on Nano-Cu/ZIF-8 Capped with Water-Soluble Pillar[6]arene and Methylene Blue Host–Guest Complexations
Source: Molecules. 2021 Jun 25;26(13):3878. doi: 10.3390/molecules26133878 (PMC8271439; doi:10.3390/molecules26133878)
Supplement: Supplementary file 1 [file molecules-26-03878-s001.zip › molecules-1263626-supplementary.pdf]

Supplementary Materials

A supramolecular photosensitizer system based on nano-Cu@ZIF-8 capped with water-soluble pillar[6]arene and methylene blue host–guest complexations

Chenhao Hu[1,+], Yueyuan Yu[1,+], Shuang Chao[1], Huidan Zhu,[2] Yuxin Pei,[1] Lan Chen[2*] and Zhichao Pei [1*]

[1] Shaanxi Key Laboratory of Natural Products & Chemical Biology, College of Chemistry and Pharmacy, Northwest A&F University, Yangling, Shaanxi 712100, P. R. China
[2] Analysis Center of College of Science & Technology, Hebei Agricultural University, Huanghua, Hebei 061100, P. R. China
***** Correspondence: peizc@nwafu.edu.cn.; Tel.: +86 2987091196 ; chenlan19860303@126.com.; Tel.: +86 3175605225
+ These authors contributed equally to this work.

Contents

1. Materials and methods

2. Characterization of the compounds

3. UV-vis and NMR studies of host-guest complexation of WP6 and MB

4. Cu/ZIF-8 Dynamic light scattering

5. Powder X-ray characterization for their materials

6. Zata potential studies of OCZWM

7. Scanning electron microscope (SEM)

8. References

1. Materials and methods

All commercially available starting materials and solvents were of reagent grade and dried prior to use. Chemical reactions were monitored with thin-layer chromatography using precoated silica gel 60 (0.25 mm thickness) plates. Flash column chromatography was performed on silica gel 60 (0.040-0.063 mm). Optical rotations were measured at the sodium D line at ambient temperature. ^{1}H and ^{13}C spectra were recorded with a 500 MHz instrument at 298K in DMSO-d6, CD_3OD, $CDCl_3$, using the residual signals from DMSO-d6 (^{1}H: δ = 2.50 ppm) , CD_3OD (^{1}H: δ = 3.34 ppm) and $CDCl_3$ (^{1}H: δ = 7.27 ppm) as internal standard.

2. Characterization of the compounds

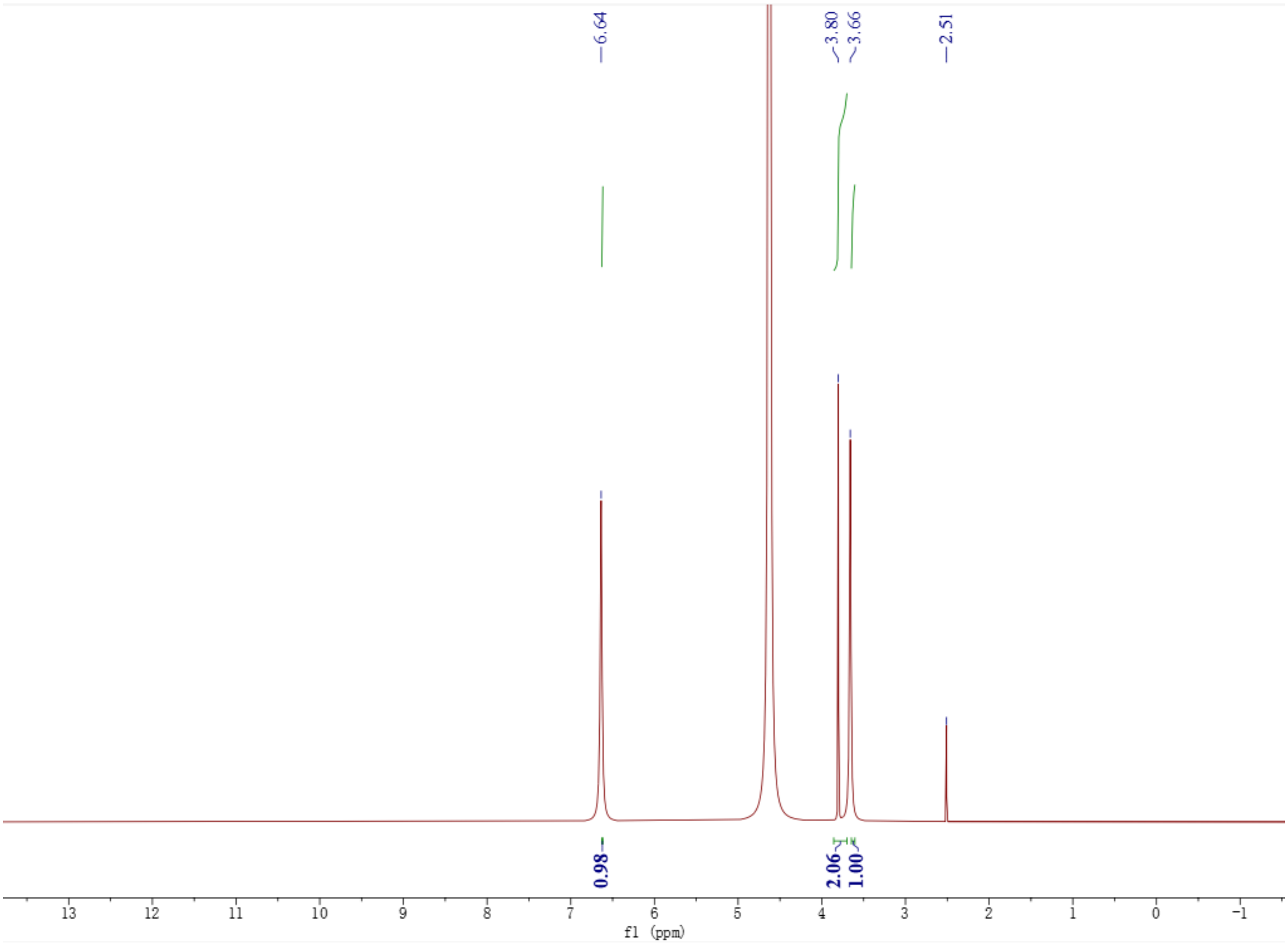

Figure S1. ^{1}H NMR spectrum (400 MHz, D_2O) of **WP6.**

3. UV-vis and NMR studies of host-guest complexation of WP6 and MB

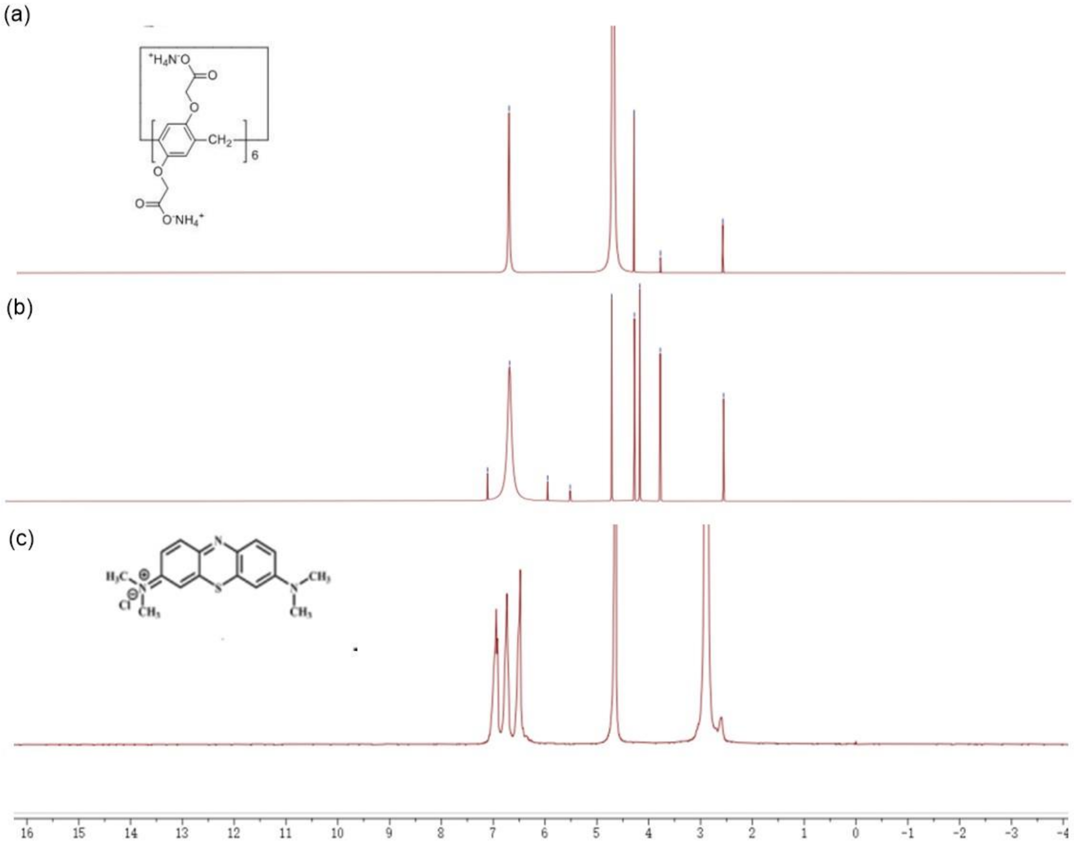

Figure S2. ^{1}H NMR spectra (400 MHz, D_2O, 298 K): (a) WP6 (10.00 mM), (b) WP6:MB = 1:1; (c) MB (10.00 mM).

4. Cu/ZIF-8 Dynamic light scattering

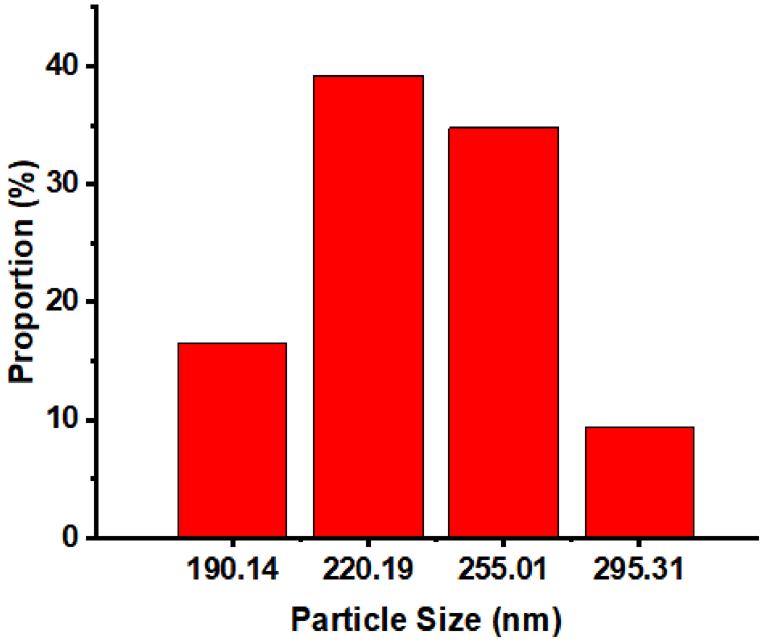

Figure S3. DLS data of Cu/ZIF-8

5. Powder X-ray characterization for their materials

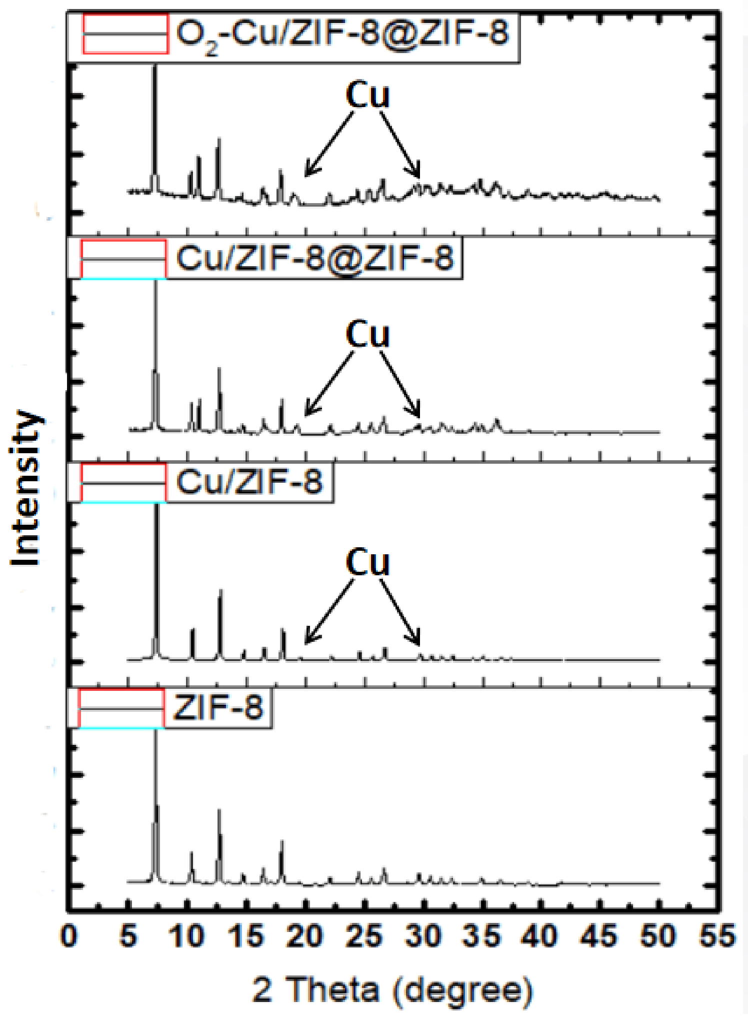

Figure S4. PXRD pattems of different materials

6. Zata potential studies of OCZWM

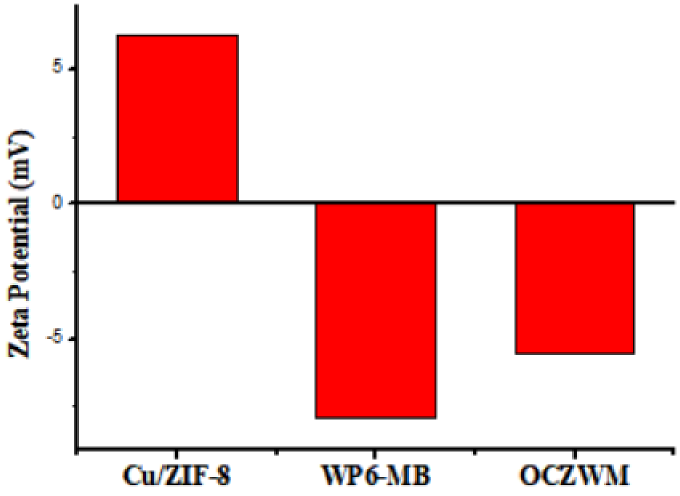

Figure S5. Zata potential studies of OCZWM

7. Scanning electron microscope (SEM)

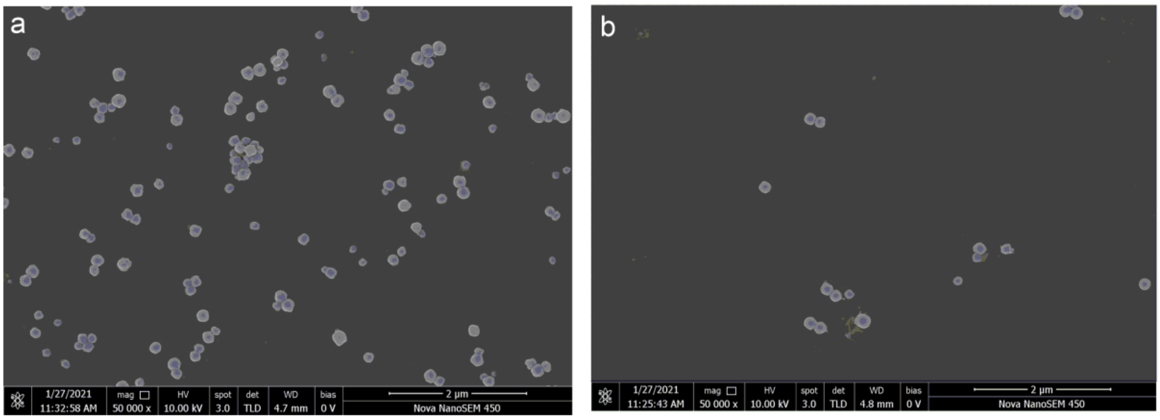

Figure S6. SEM images of Cu/ZIF-8 (a), Cu/ZIF-8@ZIF-8. The scale bar is 2 µm

8. References

S1. X. Hu, Z. Chen, L. Chen, L. Zhang, J. Hou and Z. Li, Chem. Commun., 2012, 48, 10999-11001.

S2. L. Shangguan, Q. Chen, B. Shi and F. Huang, Chem. Commun., 2017, 53, 9749-9752.

S3. D. Xia, G. Yu, J. Li and F. Huang, Chem. Commun., 2014, 50, 3606-3608.
